# The impact of triclosan on the spread of antibiotic resistance in the environment

**DOI:** 10.3389/fmicb.2014.00780

**Published:** 2015-01-15

**Authors:** Daniel E. Carey, Patrick J. McNamara

**Affiliations:** Department of Civil, Construction and Environmental Engineering, Marquette University, Milwaukee, WI, USA

**Keywords:** triclosan, antimicrobial resistance, antibiotic resistance, biosolids, wastewater

## Abstract

Triclosan (TCS) is a commonly used antimicrobial agent that enters wastewater treatment plants (WWTPs) and the environment. An estimated 1.1 × 10^5^ to 4.2 × 10^5^ kg of TCS are discharged from these WWTPs per year in the United States. The abundance of TCS along with its antimicrobial properties have given rise to concern regarding its impact on antibiotic resistance in the environment. The objective of this review is to assess the state of knowledge regarding the impact of TCS on multidrug resistance in environmental settings, including engineered environments such as anaerobic digesters. Pure culture studies are reviewed in this paper to gain insight into the substantially smaller body of research surrounding the impacts of TCS on environmental microbial communities. Pure culture studies, mainly on pathogenic strains of bacteria, demonstrate that TCS is often associated with multidrug resistance. Research is lacking to quantify the current impacts of TCS discharge to the environment, but it is known that resistance to TCS and multidrug resistance *can* increase in environmental microbial communities exposed to TCS. Research plans are proposed to quantitatively define the conditions under which TCS selects for multidrug resistance in the environment.

## INTRODUCTION

The World Health Organization warns that we may enter a post-antibiotic era in the twenty-first century due to the spread of antibiotic resistance (World Health Organization [WHO], 2014). Antibiotic resistance is defined as the ability of bacteria to survive a concentration of antibiotics that typically inhibits growth of the majority of other bacteria (Russell, 2000). Antibiotics are extensively used in medicine to treat bacterial infections in humans and animals, and are widely used in agriculture to promote animal growth (Khachatourians, 1998; Kümmerer, 2004). Each year, in the United States (U.S.) alone, over two million people are infected by antibiotic resistant bacteria, leading to more than 25,000 deaths, and $50 billion spent managing antibiotic resistance (Centers for Disease Control and Prevention [CDC], U.S. Department of Health, and Human Services, 2013). The associated cost continues to increase as bacteria acquire mechanisms to fight against the antibiotics that are typically employed (Levy and Marshall, 2004).

In addition to antibiotics, synthetic antimicrobial agents are also pervasive in households and hospitals, mainly for disinfection and sanitation purposes. The term “antimicrobial” has been used to describe a broad range of compounds, including antibiotics that destroy or inhibit microorganisms (McDonnell and Russell, 1999; Kümmerer, 2004). For this paper, triclosan (TCS), which is not derived naturally, is referred to as an antimicrobial. Compounds produced or derived from microorganisms used *in vivo* to treat bacterial infections in eukaryotes (e.g., erythromycin, tetracycline, ciprofloxacin, etc.) will be referred to as antibiotics (even though antibiotics are a subset of antimicrobials).

Triclosan is widely used for personal hygiene and disinfection purposes; in fact, 350 tons were produced for commercial use in the European Union in 2002. Based on 1998 records from the Environmental Protection Agency, approximately 500–5000 tons were produced in the U.S., and the industry has reported growth (Singer et al., 2002; Heidler and Halden, 2007; Fang et al., 2010; Venkatesan and Halden, 2014). With these approximations, it is estimated that 1 kg of TCS is produced for every 3 kg of antibiotics produced (Food and Drug Administration [FDA], 2011; Department of Health and Human Services [DHHS], 2012). TCS is found in a wide range of consumer products including hand soap, toothpaste, deodorant, surgical scrubs, shower gel, hand lotion, hand cream, and mouthwash (Bhargava and Leonard, 1996; Jones et al., 2000).

Because of its wide use, TCS is found in many natural and engineered environments, including surface water, wastewater, soil, drinking water, wastewater treatment plants (WWTPs), biosolids, landfills, and sediments (Singer et al., 2002; Miller and Heidler, 2008; Benotti et al., 2009; Kumar et al., 2010; Xia et al., 2010; Welsch and Gillock, 2011; Bedoux et al., 2012; Mavri et al., 2012). As TCS is commonly used in oral consumer products, it is widely found in human urine. In a survey of 181 pregnant women in an urban multiethnic population in Brooklyn, NY, TCS was found in 100% of urine samples (Pycke et al., 2014). In a geographically broader U.S. survey, 75% of people were found to have TCS in their urine (Calafat et al., 2008).

At application concentrations (0.1–0.3 w/v% or approximately 1,000–3,000 mg/L in hand soaps), TCS induces cell damage that causes cell contents to physically leak out of the membrane (Villalaín et al., 2001). At concentrations lower than 1 mg/L, TCS serves as an external pressure to select for TCS resistance as well as antibiotic resistance in many types of bacteria (Russell, 2000; Schweizer, 2001; Poole, 2002; Chapman, 2003; Yazdankhah et al., 2006; Birosová and Mikulásová, 2009; Saleh et al., 2011; Halden, 2014). At low concentrations, TCS interacts with physiological targets, and these interactions lead to numerous resistance mechanisms that are reviewed below (Chuanchuen et al., 2001; Bailey et al., 2008; Yu et al., 2010; Condell et al., 2012). In some cases, the mechanisms that convey resistance to TCS simultaneously confer resistance to more than one class of antibiotics (Poole, 2002; Alanis, 2005).

The wide use of TCS leads to concern about its potential to aid in the spread of antibiotic resistance (Russell, 2000; Kümmerer, 2004; Saleh et al., 2011). TCS exposure that leads to TCS resistance and antibiotic resistance has been widely reported, but the majority of these studies pertain to pure cultures of specific bacterial strains, and in most cases, pathogenic strains. This line of research is logical because antibiotic resistant pathogens are of greatest concern to public health. TCS might also impact the spread of resistance in environmental microbial communities as approximately 1.1 × 10^5^ to 4.2 × 10^5^ kg of TCS are distributed to the environment annually through WWTPs in the U.S. (Heidler and Halden, 2007). Studies on pure culture isolates provide insight into the *potential* impacts of TCS on antibiotic resistance in environmental bacterial communities. The important question then becomes: does TCS select for antibiotic resistance in these complex microbial communities?

Many engineered and natural processes are driven by microbes, and TCS is designed to impact microbes in homes and hospitals. Following discharge to the environment, the antimicrobial properties of TCS can impact complex microbial communities found in engineered and environmental systems. TCS has been linked to altering microbial community structure or function in wastewater operations, such as activated sludge and anaerobic digestion (Stasinakis et al., 2008; McNamara et al., 2014). Likewise, TCS can alter diversity and biofilm development in freshwater biofilms in receiving streams (Johnson et al., 2009; Proia et al., 2011; Lubarsky et al., 2012). In soils, TCS impacts respiration rates and denitrification, and enriches for species capable of dehalogenation (Butler et al., 2011; McNamara and Krzmarzick, 2013; Holzem et al., 2014). TCS induces responses in microbial communities, but the TCS concentrations that inhibit function are not often found in these complex microbial communities. At environmental concentrations, TCS is more likely to exert a stress that propagates resistance than to exert a stress that functionally inhibits complex microbial communities.

The purpose of this manuscript is to review the state of knowledge regarding the impact of TCS on antibiotic resistance in environmental systems and identify critical research questions that need to be addressed to better understand the impact of TCS-derived resistance in the environment on public health. This review describes TCS resistance and cross-resistance in pure cultures, and then considers the comparatively smaller amount of literature that addresses how TCS impacts antibiotic resistance in engineered environments containing complex microbial communities. Engineered environments are of prime interest because they contain TCS, bacteria, and resistance genes that can be subsequently dispersed to terrestrial soils and surface waters with the possibility of negative public health consequences (Pruden et al., 2006, 2012; Baquero et al., 2008; Cha and Cupples, 2009; Ghosh et al., 2009; Munir et al., 2010; LaPara et al., 2011; Ma et al., 2011; Burch et al., 2014; Yang et al., 2014).

## GENETIC TARGETS OF TRICLOSAN

In 1998, TCS was first described by McMurry et al. (1998b) to have a specific target in *Escherichia coli*. At 1 mg/L, approximately 1000-fold lower than the application concentration, TCS inhibits FabI, an enoyl-acyl carrier protein reductase (ENR). The FabI protein catalyzes the elongation cycle in the synthesis of fatty acids, an essential process for cell viability (Bergler et al., 1996; Massengo-Tiassé and Cronan, 2008, 2009). Prior to McMurry et al.’s (1998b) report, low concentrations of TCS were assumed to have minimal effects on cell viability.

Up-regulation of *fabI* is a response mechanism which may overcome the effects of intracellular TCS (Condell et al., 2012; Yu et al., 2012; Sheridan et al., 2013). Bacteria can up-regulate and down-regulate many more genes in response to TCS, although it can be difficult to determine which expression changes are casual. No universal response has been observed; however, many bacteria respond to some degree with the up-regulation of transport proteins and membrane bound proteins (Bailey et al., 2008; Chuanchuen and Schweizer, 2012).

## TCS RESISTANCE IN PURE CULTURES

The most common resistance mechanisms based on pure culture studies are target site modification, membrane resistance, and efflux. The following sections briefly review resistance mechanisms to TCS and describe their impact on cross-resistance; a comprehensive review of TCS resistance mechanisms can be found by Schweizer (2001).

### FABI MODIFICATION OR REPLACEMENT

Target site modification is a resistance mechanism that involves a genetic alteration to the target site that reduces the effect of an inhibitory chemical (Hooper, 2005). Modification of TCS target site FabI is a common resistance mechanism observed in pure cultures. Mutation occurs whereby single or multiple amino acids are changed in the *fabI* gene, resulting in TCS-resistant FabI proteins (Brenwald and Fraise, 2003; Yu et al., 2010). Ciusa et al. (2012) suggested a resistance mechanism whereby an allele of a *fabI* gene is located on a mobile genetic element and transposed into *Staphylococcus aureus*. The presence of the *fabI* allele together with the intrinsic *fabI* gene increased the concentration of the FabI protein through heterologous duplication and increased bacterial tolerance to TCS. Alternatively, ENR isoenzymes, which perform similar functions to FabI, including FabL, FabK, and FabV, have been identified in TCS-resistant bacteria (Massengo-Tiassé and Cronan, 2009). These isoenzymes are naturally found in some strains of bacteria. In fact, FabV has been found to functionally replace FabI, rendering *Pseudomonas aeruginosa* 2,000 times more resistant to TCS as seen by an increase in minimum inhibitory concentration (MIC; Zhu et al., 2010). Similarly, FabK replaces function for FabI in *Streptococcus pneumonia*, leading to increased tolerance to TCS (Heath et al., 2000), and FabL expression leads to increased resistance to TCS in *Bacillus subtilis* (Heath et al., 2000).

With respect to multidrug resistance, FabI alteration or replacement may specifically produce resistance to isoniazid, an important agent for the treatment of tuberculosis, which also targets FabI (Ciusa et al., 2012). However, FabI alterations are not generally known to cause resistance to other antibiotics. This type of resistance in environmental communities would not likely pose a threat to public health through increased multidrug resistance.

### MEMBRANE ALTERATION

Modifications through changes to the outer membrane is a less-studied TCS resistance mechanism in bacteria. Champlin et al. (2005) concluded that outer membrane properties were responsible for low-level resistance to hydrophobic antimicrobials and antibiotics. The researchers compared *P. aeruginosa* strains that possessed highly refractory outer cell envelopes to strains that had highly permeable outer cell envelopes and discovered that the outer membrane properties conferred intrinsic resistance to TCS up to 256 mg/L. Tkachenko et al. (2007) suggested that TCS exposure could induce a genetic response which increases the concentration of branched chain fatty acids in the cell membrane in *S. aureus*; the membrane thereby sequesters the chemical agent and stops it from passing into the cell, preventing physiological disruption inside of the cell.

Outer membrane impermeability is a potential mechanism for cross-resistance to antibiotics. Particularly, non-specific rejection of hydrophobic chemicals could be a mechanism for resistance to TCS and other antibiotics that may be found in the environment.

### EFFLUX PUMPS

Efflux pumps are often associated with multidrug resistance, which is a public health concern. Active efflux, whereby a bacterium physically removes a constituent from its intracellular space by pumping the constituent across the membrane and back into the environment, is an effective mechanism against a wide range of antimicrobials and antibiotics, including TCS (Kern et al., 2000; Levy, 2002). The AcrAB efflux pump is responsible for efflux of TCS in *E. coli* and *Salmonella enterica* (McMurry et al., 1998a; Webber et al., 2008). Non-specific multidrug efflux pumps (e.g., *mex* proteins) confer resistance to TCS as well as other antibiotics in *P. aeruginosa* and *Rhodospirillum rubrum* (Chuanchuen et al., 2001; Pycke et al., 2010a,b). Most non-specific efflux pumps are capable of expulsing antibiotics. Thus, in cases where bacteria acquire non-specific efflux pumps through horizontal gene transfer after exposure to TCS, the bacteria would likely acquire resistance to antibiotics as well. In some cases specific efflux pumps confer resistance to TCS. TriABC-OpmH is a TCS-specific efflux pump in *P. aeruginosa* that is not known to expel other compounds such as antibiotics (Mima et al., 2007).

### TRICLOSAN AND CROSS-RESISTANCE TO ANTIBIOTICS

**FIGURE 1:**
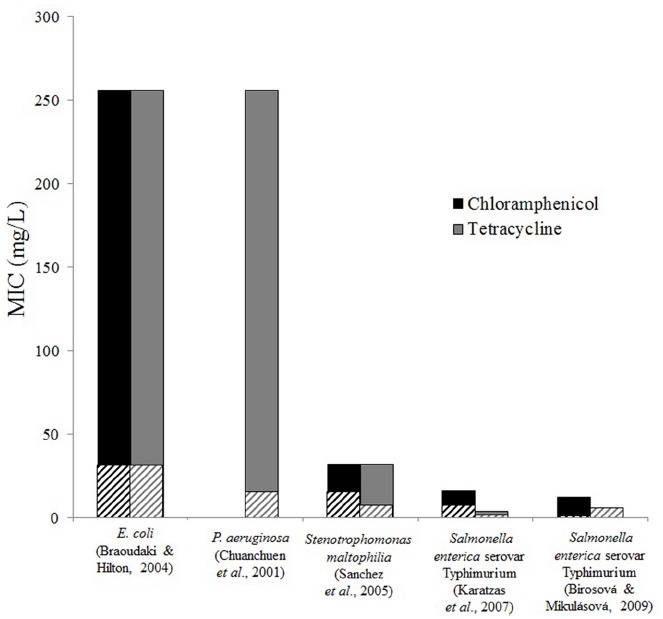
**Triclosan exposure increases resistance to antibiotics.** Minimum inhibitory concentrations of chloramphenicol and tetracycline for control strains (striped bars) and TCS adapted strains (solid bars) are shown from various studies and bacteria. Differences were observed in most cases, however, no difference was found for tetracycline resistance for *Salmonella enterica* in the study by Birosová and Mikulásová (2009). Chloramphenicol resistance was not tested in *Pseudomonas aeruginosa* (Chuanchuen et al., 2001).

Resistance to TCS, incurred by exposure to TCS, can directly affect resistance to antibiotics. Cross-resistance has been tested for a wide range of antibiotics following exposure to TCS. Chloramphenicol and tetracycline are two antibiotics commonly included in antibiotic cross-resistance experiments. In studies done on *E. coli* and *P. aeruginosa*, resistance to chloramphenicol and tetracycline increased 10-fold following TCS exposure (Figure 1). Increased antibiotic resistance in *Stenotrophomonas maltophilia* and *Salmonella enterica* serovar Typhimurium following TCS exposure was also observed, but the increase was less severe. Cross resistance in *P. aeruginosa* (Chuanchuen et al., 2001), *Stenotrophomonas maltophilia* (Sanchez et al., 2005), and *Salmonella enterica* serovar Typhimurium (Karatzas et al., 2007) were attributed to efflux systems. Resistance mechanisms were not directly investigated in the studies on *E. coli* (Braoudaki and Hilton, 2004) and *Salmonella enterica* serovar Typhimurium (Birosová and Mikulásová, 2009), however *acrAB* genes, which encode for efflux, are known to confer resistance to TCS, chloramphenicol, and tetracycline in both of these species (Karatzas et al., 2007). These findings highlight a main concern regarding the widespread dissemination of TCS, i.e., that TCS exposure can spread multidrug resistance.

Triclosan resistance and antibiotic resistance have been found together in clinical isolates. In a survey of 732 clinical isolates of *Acinetobacter baumannii* from hospitals, 3% of isolates were found to have reduced susceptibility to TCS (MIC > 1 mg/L; Chen et al., 2009). Those isolates which could tolerate higher than 4 mg/L also had increased tolerance to amikacin, tetracycline, levofloxacin and imipenem. Clinical isolates of *S. aureus*, which had MICs to TCS between 0.025 and 1 mg/L, were resistant to multiple antibiotics (Suller and Russell, 2000). Some, but not all, of the strains showed increased resistance to gentamicin, erythromycin, penicillin, rifampicin, fusidic acid, tetracycline, methicillin, mupirocin, and streptomycin. In some strains TCS resistance was stable when sub-culturing was performed in a TCS-free medium. In other strains TCS resistance was lost when the strain was propagated for 10 days in TCS-free media, indicating that the presence of TCS can select for resistance that is not regularly expressed. This finding implies that removing TCS from environmental systems through improved treatment processes or reduced consumer usage could lead to a decrease in TCS resistance. Research should be conducted to specifically test the impacts of removing TCS on TCS-derived resistance in complex microbial communities.

Conditions that perpetuate resistance to TCS frequently result in cross-resistance to antibiotics. TCS resistant *Salmonella enterica* serovar Typhimurium strains were selected by daily sub-culturing of TCS-exposed cultures and increasing TCS concentrations in media from 0.05 to 15 mg/L over 15 days (Karatzas et al., 2007). The TCS MIC in the resulting strains increased from 0.06 mg/L to as high as 128 mg/L, and the strains were also more resistant to ampicillin, tetracycline, and kanamycin. The authors concluded that the overexpression of the *acrAB* efflux pump was likely involved in the increased tolerance to TCS and antibiotics. In another study, TCS selected for ciprofloxacin resistant mutants in *Salmonella enterica* serovar Typhimurium when exposed to 0.5 mg/L of TCS (Birosová and Mikulásová, 2009). These studies, along with the concentrations of TCS found in the environment, imply that TCS could select for bacteria in environmental communities that have efflux pumps.

Efflux is a common method of resistance, but the specific efflux system used and the resulting cross-resistance profile can vary between species. In *P. aeruginosa*, MexAB-OprM, MexCD-OprJ, and MexEF-OprN, contribute to TCS resistance (Chuanchuen et al., 2001). Exposure to TCS selected for up-regulation of these efflux systems due to mutations in the regulatory gene, *nfxB*, which increased the tolerance to tetracycline, ciprofloxacin, trimethoprim, erythromycin, and gentamicin. In some cases the TCS resistant strains could tolerate up to 500-fold higher antibiotic concentrations than the non-TCS resistant strains. Strains which lacked these efflux systems showed increased sensitivity to antibiotics. In the opportunistic pathogen *Stenotrophomonas maltophilia*, TCS binds to the repressor SmeT, allowing expression of an efflux pump, SmeDEF (Hernández et al., 2011). Expression of this efflux pump following exposure to TCS resulted in increased resistance to the antibiotics ciprofloxacin, norfloxacin, nalidixic, and ofloxacin. Sanchez et al. (2005) also found that TCS-resistant mutants of *Stenotrophomonas maltophilia* (tolerant up to 64 μg/L of TCS) overexpress SmeDEF. These mutants had an increased tolerance to tetracycline, chloramphenicol, and ciprofloxacin. Even though SmeDEF is intrinsically contained in the genome of *Stenotrophomonas maltophilia,* TCS exposure selected for up-regulation of this efflux pump which increased antibiotic resistance.

In addition to variances between genera, cross-resistance varies within genera. TCS-adapted *E. coli* O157:H7 exhibited increased resistance to chloramphenicol, tetracycline, amoxicillin, amoxicillin/clavulanic acid, trimethoprim, benzalkonium chloride, and chlorhexidine, while TCS-adapted *E. coli* O55 exhibited resistance to only trimethoprim (Braoudaki and Hilton, 2004).

Although most evidence supports the notion that TCS increases resistance to antibiotics, this is not necessarily true for all classes of antibiotics. In one case, TCS-resistant mutants of *Salmonella enterica* were more (or no less) susceptible to antibiotics (Rensch et al., 2013). *Salmonella enterica* that were selected to have overexpression of *fabI* or a *fabI* mutation had increased susceptibility to the aminoglycoside antibiotics kanamycin and gentamicin.

The cross-resistance profiles vary among the bacteria surveyed in this review, and other types of bacteria yet to be studied are likely to have unique cross-resistance profiles. While resistance profiles vary, the overarching theme is the same: resistance to TCS can yield cross-resistance to multiple antibiotics. Given that TCS is not an antibiotic, resistance to TCS alone is not a public health threat. TCS-derived proliferation of multidrug resistant bacteria, however, could be a severe threat to public health. These pure culture studies indicate that TCS is likely to select for multidrug resistant bacteria above a critical concentration. In environmental communities, such as anaerobic digesters or sediments, TCS is found at 2- to 1000-fold higher concentrations than any given antibiotic (McClellan and Halden, 2010). Is TCS selecting for resistant bacteria in the environment? The role of TCS on the selection of antibiotic resistance genes and multidrug resistance genes in the environment needs to be quantified to determine what steps, if any, are necessary for protecting public health.

## TRICLOSAN-DERIVED RESISTANCE IN COMPLEX ENVIRONMENTAL COMMUNITIES

Environmental systems, including WWTPs and sediments, represent the most likely sites for TCS resistance to develop because of the high abundance of TCS and high density of bacteria. Wastewater treatment systems should be given special focus because they contain and discharge TCS and resistance genes to the environment. To understand the role of TCS and the remaining research gaps, the fate of TCS in the environment is summarized to highlight locations of prime interest, and the state of knowledge regarding TCS and resistance in complex microbial communities is assessed.

### FATE OF TRICLOSAN

Triclosan is discharged into the environment with treated liquid and solid effluents from WWTPs. In the U.S. alone, WWTPs are estimated to receive approximately 100 tons of TCS each year, but the prevalence of TCS in treated effluent is not restricted to U.S. facilities. A survey of WWTPs in Germany found TCS in treated effluents at concentrations ranging from 1 × 10^–5^ to 6 × 10^–4^ mg/L (Bester, 2005). The concentrations of TCS and its aerobic degradation products in receiving waters were less than 3 × 10^–6^ mg/L. A study of eight WWTPs in Switzerland revealed that, on average, 6% of the influent TCS was found to discharge with the effluent water at concentrations of 4.2 × 10^–5^ to 2.13 × 10^–4^ mg/L (Singer et al., 2002); these receiving streams had concentrations at 1.1 × 10^–5^ to 9.8 × 10^–5^ mg/L. A more recent study found TCS in WWTP effluents at 9.7 × 10^–5^ mg/L, and in nearby sediments at 0.018 mg/kg (Blair et al., 2013). Several other studies have found TCS in surface water in concentrations ranging from <2 × 10^–7^ mg/L up to 0.022 mg/L (Bedoux et al., 2012).

Triclosan that is discharged with liquid effluent often partitions to sediments. Miller and Heidler (2008) found that TCS accumulated in sediments near WWTP outfalls for approximately 50 years, and similar results were found by other researchers (Buth et al., 2010; Anger et al., 2013). Sediment concentrations have been found at 53 mg/kg (Chalew and Halden, 2009). TCS is prevalent in liquid effluents and abundant in sediments, but this discharge route does not account for the majority of TCS that enters the environment. One study estimated that 0.24 kg/day of TCS are released with liquid effluent, but 5.37 kg/day are released with the treated residual solids from a midsized WWTP (Lozano et al., 2013).

Indeed, nearly half (or even higher) of the influent TCS load to WWTPs is captured by solids following sorption (Heidler and Halden, 2007; Lozano et al., 2013). The concentration of TCS in biosolids is often much higher than in aqueous systems because of the hydrophobic nature of TCS (Heidler and Halden, 2008). A nationwide U.S. survey of TCS in biosolids found the median concentration in treated biosolids to be 3.9 mg/kg and the maximum level was 133 mg/kg (United States Environmental Protection Agency [USEPA], 2009). The high levels found in biosolids can lead to high levels in soils when biosolids are land applied. TCS was found in biosolids-amended soils which had been receiving biosolids for 33 years (Xia et al., 2010). The concentrations in the soil ranged from approximately 1 mg/kg in the first 15 cm of soil to less than 0.1 mg/kg at a depth of 60–120 cm. The half-life of TCS in soil under aerobic conditions was 104 days, and TCS is even more persistent under anaerobic conditions (McAvoy et al., 2002; Ying et al., 2007). These fate data, along with the hydrophobic nature of TCS, indicate that TCS is most likely to impact microbial communities that contain high concentrations of organic matter, including anaerobic digesters, sediments, and soils, and these communities should receive special focus when investigating TCS-derived resistance in the environment.

**FIGURE 2:**
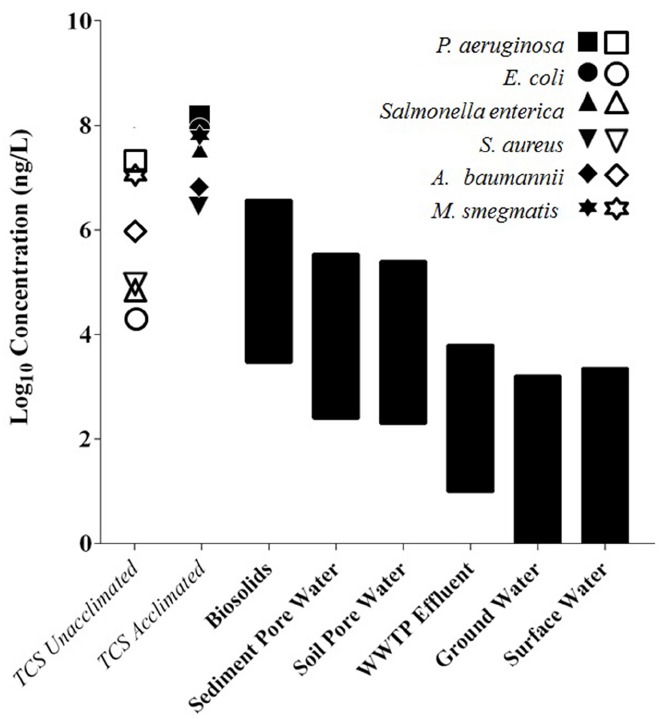
**The MIC of TCS-acclimated and TCS-unacclimated strains relative to environmental TCS concentrations.** Open symbols represent the MIC for TCS sensitive strains, while closed symbols represent the MIC for TCS adapted strains. Black bars are ranges of TCS concentrations found in each environmental setting. Biosolids concentrations were converted from mg/kg to mg/L by assuming 3% total solids in reactors that produce biosolids (McMurry et al., 1998a, 1999; Chuanchuen et al., 2001; Slater-Radosti et al., 2001; Fan et al., 2002; Yazdankhah et al., 2006; Karatzas et al., 2007; Mima et al., 2007; Tkachenko et al., 2007; Bailey et al., 2008; Webber et al., 2008; Chalew and Halden, 2009; Chen et al., 2009; McClellan and Halden, 2010; Yu et al., 2010; Saleh et al., 2011; Bedoux et al., 2012).

The range of TCS concentrations found in the environment is depicted in Figure 2 along with the MIC of TCS-acclimated and TCS-unacclimated pathogenic strains of bacteria. The concentrations in the biosolids and sediments are higher than the MICs of TCS-sensitive strains, indicating that TCS-sensitive strains would not thrive in these environments and TCS-resistant strains may be present. The MICs of TCS-acclimated strains, however, are higher than the current environmental TCS concentrations and could tolerate an increase in TCS concentrations. A future increase in TCS concentrations may select for resistance rather than functionally inhibit complex microbial communities. This figure indicates that biosolids and sediment environments with high TCS concentrations likely have TCS-resistant bacteria, but this figure does not indicate the level of TCS required to select or enrich for resistance in the environments with lower TCS concentrations. What happens when TCS is below the MIC? Certainly environments with very high levels of TCS will have TCS-resistant strains, but do environments with TCS concentrations below the MIC of acclimated strains select for resistance? What concentration of TCS is required to select for resistance in various environmental communities? These questions represent critical research gaps. By answering these questions with further research we can determine if and where TCS is selecting for resistance. Research plans are outlined in the final section to address these questions.

### TRICLOSAN RESISTANCE IN COMPLEX MICROBIAL COMMUNITIES

Bacteria with resistance to TCS are found in the environment, and experiments have been performed to determine whether TCS could be the cause for resistance. Drury et al. (2013) constructed artificial streams to control for other selective pressures such as antibiotics. The artificial streams were inoculated with approximately 8 mg/L of TCS. Over 34 days, the relative abundance of benthic bacteria which were able to be cultivated in 16 mg/L of TCS in agar climbed from 0 to 14%. In a similar study, TCS was added to artificial stream mesocosms at 1 × 10^–4^, 5 × 10^–4^, 1 × 10^–3^, 5 × 10^–3^, and 1 × 10^–2^ mg/L, and resistance to TCS significantly increased in bacterial populations exposed to TCS concentrations over 5 × 10^–4^ mg/L (Nietch and Quinlan, 2013). This study was conducted at environmentally relevant concentrations, and suggested that TCS exposure leads to TCS-resistance. Middleton and Salierno (2013) discovered that TCS resistance was detected in 78.8% of fecal coliform samples from streams receiving wastewater, and 89.6% of these samples were resistant to four classes of antibiotics. *Escherichia*, *Enterobacter*, *Serratia*, and *Citrobacter* were also found in the stream with resistance to TCS and multiple antibiotics. This study investigated real-world surface water samples which are implicitly associated with many uncontrolled variables. Accordingly, it infers, but does not prove, that TCS may be an external stressor that results in increased abundance of resistance genes.

Studies on the impacts of TCS on anaerobic digesters, where TCS is of highest abundance, are lacking. Lab-scale studies revealed that TCS can affect multidrug resistance genes in anaerobic bioreactors. McNamara et al. (2014) found that TCS at 500 mg/kg selected for *mexB* in lab-scale anaerobic digesters inoculated with cow manure. In anaerobic digesters that were seeded with municipal biosolids, 500 mg/kg did not select for* mexB*, but methane production was inhibited. It is not yet known if anaerobic communities need to carry resistance genes in order to maintain function at these high TCS levels. The findings indicated that the microbial community structure, in addition to the concentration of TCS, influences the selection of resistance genes. Also, this research demonstrated that TCS can select for resistance, but does selection happen at environmental concentrations of TCS? Similarly, in activated sludge mesocosms, TCS selected for *tetQ* at 0.3 mg/L of TCS (Son et al., 2010). These two wastewater studies found a correlation between the presence of a resistance gene and TCS, but each study only investigated a single gene. A much more thorough research effort is required to determine the breadth of genes, with a special emphasis on multidrug resistance genes, that are selected for when environmental concentrations of TCS are applied to the complex microbial communities found in WWTPs.

It is also possible that TCS-resistant bacteria are formed in premise plumbing which can feed into municipal WWTPs. In a sink drain biofilm, TCS was shown to affect the bacterial population structure when a 0.2% (∼2000 mg/L) solution of soap containing TCS was pumped over the biofilm (Mcbain et al., 2003). Overall bacterial diversity was reduced and several TCS-resistant bacteria related to *Achromobacter xylosoxidans* increased in abundance, while other species including aeromonads, bacilli, chryseobacteria, klebsiellae, stenotrophomonads, and *Microbacterium phyllosphaerae* were reduced. TCS in a drain following consumer usage may result in resistant bacteria which are then sent to WWTPs. Research is needed to determine if these bacteria survive in the sewer system and whether these resistant bacteria influence the resistance profile in WWTPs.

These studies show that TCS in the environment could select for resistance genes. It seems likely that TCS resistance coincides with TCS-derived cross-resistance to antibiotics in the environment, but further studies are required to validate this point.

## RESEARCH GAPS AND CONCLUSIONS

It is noted that pathogenic bacteria, such as *S. epidermidis*, are less susceptible to TCS today than they were in the past (Skovgaard et al., 2013). Although resistance to TCS alone is not a threat to human health, antibiotic resistance is a major public health concern. TCS is widespread throughout the environment, but the direct role of TCS on antibiotic resistance in environmental systems is not yet defined. Four specific research questions, which are outlined below, need to be answered to identify the role of TCS on antibiotic resistance in environmental systems and ultimately determine the impact on human health.

### IDENTIFY THE ROLE OF TRICLOSAN ON ANTIBIOTIC RESISTANCE IN ENVIRONMENTAL SYSTEMS

#### What is the threshold concentration of TCS that triggers resistance?

Triclosan is found at a wide range of concentrations in a wide range of environments (see Figure 2), and previous work found that TCS *can* select for a resistance gene in a complex microbial community (Son et al., 2010; McNamara et al., 2014). Moving forward it is important to determine the concentrations of TCS that trigger an increase in antibiotic resistance genes. Answering this question will also help address the question framed by the lack of data in Figure 2, i.e., what is the effect of TCS concentrations below the MIC? Do low levels of TCS select for resistance? Chronic exposure experiments using lab mesocosms should be performed at a range of steady-state TCS concentrations. In most real world cases, TCS levels will slowly increase, and lab experiments should be designed to reflect this slow loading rate. TCS levels should be slowly increased over time and held constant during steady-state operation of the mesocosm to determine the concentration of TCS that sustains changes in antibiotic resistance profiles. Metagenomics can be used with the Antibiotic Resistance Genes Database (Liu and Pop, 2009) to determine how the concentration of TCS impacts the relative abundance of antibiotic resistance genes. Additionally, qPCR can be employed to quantify changes in resistance gene abundance. After completion of these experiments we will have a better understanding about the concentrations of TCS that trigger increases in antibiotic resistance genes.

#### What is the role of the microbial community composition on TCS-derived antibiotic resistance?

Previous work revealed that the same concentration of TCS can lead to different impacts on the abundance of a resistant gene depending on the microbial community (McNamara et al., 2014). Experiments outlined in the question above should be performed on several different microbial communities. For example, communities found in river sediments, soils, and anaerobic digesters, should be investigated, and experiments should also be performed on several different communities from each type of environment. Wastewater communities can vary widely in their structure and so could the impact of TCS on resistance in these communities. Mesocosms should be inoculated with biosolids from several different cities to quantify how the same TCS concentrations impact the antibiotic resistance profiles of different communities. Is there a universal TCS concentration that is of concern in anaerobic digester communities, in sediments, or in soils? Illumina sequencing on 16S rRNA genes should be performed as well to determine if a link exists between certain microbes in a community and the TCS-impacted resistance profile.

#### What is the impact of TCS on resistance profiles in environments that are also perturbed by antibiotics?

Some resistance mechanisms, mainly efflux pumps, which are triggered by TCS are also triggered by antibiotics. In environments perturbed by TCS, antibiotics are also present (e.g., McClellan and Halden, 2010). Does the presence of TCS impact the acquisition of antibiotic resistance genes through horizontal gene transfer when antibiotics are already present? In other words, if TCS were not in these environments would the resistance profile look the same? To help answer this question, mesocosms could be inoculated with complex microbial communities from environments that are not heavily impacted by antibiotics or TCS. One set of mesocosms could be amended with antibiotics and another set would be amended with antibiotics and TCS. It is important to add TCS and antibiotics at ratios typically found in the environment. Granted, this question is difficult to answer because complex microbial communities from pristine environments will have inherent differences from the communities that are typically exposed to TCS and antibiotics. Another possibility would be to use a microbial community that has been widely exposed to antibiotics but not exposed to TCS; this type of community might be readily found in countries that have not adopted wide-spread use of TCS. Molecular techniques described above could be employed to determine the added impact of TCS on antibiotic resistance gene profiles.

#### Will the abundance of resistance genes decrease if TCS concentrations decrease?

It is important to know the concentrations of TCS that select for resistance and the communities that are most vulnerable to resistance caused by TCS, but it is equally important to know if resistance caused by TCS is reversible. Mitigated use of TCS has been proposed in the U.S. in part because of the potential concerns over antibiotic resistance (Landau and Young, 2014). If there were to be a sudden decline in consumer usage, would TCS-resistance and associated multidrug resistance decrease? Experiments should be performed where TCS is slowly increased to encourage TCS-resistance and the mesocosms should be operated at steady-state with a constant supply of TCS. After the resistance profiled is determined, TCS should be removed from the system while the mesocosms are maintained under TCS-free conditions. The resistance profile can then be quantified after TCS is washed out of the mesocosms to determine if TCS-derived resistance will decrease as TCS levels decrease. This set of experiments would help to determine the potential impacts of reducing TCS from environmental systems.

### IDENTIFY THE IMPACT OF TRICLOSAN-DERIVED RESISTANCE IN THE ENVIRONMENT ON PUBLIC HEALTH

Complex microbial environments can be highly conducive for the transfer of resistance genes (Baquero et al., 2008). Locations with high densities of bacteria, such as WWTPs, produce conditions which are suitable for proliferation and exchange of resistance genes, and TCS may be serving as a selective pressure to increase the abundance of resistance genes in these communities. In a study focusing on plasmid genes found in activated sludge, a wide array of resistance genes, including genes that confer resistance to TCS in pure cultures (*mexB*, and other efflux pump homologues including *acrB* and *smeE*) were found on plasmids (Zhang et al., 2011). Research is needed to address the fate of environmentally derived resistance genes to understand how they impact human health.

The fate and transport of these resistance genes in the environment following discharge from WWTPs is not well defined. Transport of genes can occur through direct uptake of DNA (transformation), by viral infection (transduction), or by transfer of plasmids and other mobile genetic elements (conjugation); the resulting pathways for genetic transport are complicated to constrain for modeling (Baquero et al., 2008). Genetic tracking of resistance in the environment would require vast resources; using established models of viruses or bacteria may be an appropriate place to begin modeling resistance gene transport.

The rate of transfer of antibiotic resistance genes in the environment to humans is also under investigation (Viau et al., 2011; Ashbolt et al., 2013). Better understanding the threat of environmentally derived antibiotic resistance genes on human health is required to determine the role of TCS on public health. Employing quantitative microbial risk assessment for antibiotic resistance genes in environmental systems may be a useful avenue for pursuing this topic.

### Conflict of Interest Statement

The authors declare that the research was conducted in the absence of any commercial or financial relationships that could be construed as a potential conflict of interest.
